# Advances in Cancer Therapy: A Comprehensive Review of CDK and EGFR Inhibitors

**DOI:** 10.3390/cells13191656

**Published:** 2024-10-06

**Authors:** Mohammed Hawash

**Affiliations:** Department of Pharmacy, Faculty of Medicine and Health Sciences, An-Najah National University, Nablus P.O. Box 7, Palestine; mohawash@najah.edu; Tel.: +970-569939939

**Keywords:** cancer, cell lines, kinases, CDKs, EGFR, FDA, targeted therapy, cell cycle

## Abstract

Protein kinases have essential responsibilities in controlling several cellular processes, and their abnormal regulation is strongly related to the development of cancer. The implementation of protein kinase inhibitors has significantly transformed cancer therapy by modifying treatment strategies. These inhibitors have received substantial FDA clearance in recent decades. Protein kinases have emerged as primary objectives for therapeutic interventions, particularly in the context of cancer treatment. At present, 69 therapeutics have been approved by the FDA that target approximately 24 protein kinases, which are specifically prescribed for the treatment of neoplastic illnesses. These novel agents specifically inhibit certain protein kinases, such as receptor protein-tyrosine kinases, protein-serine/threonine kinases, dual-specificity kinases, nonreceptor protein-tyrosine kinases, and receptor protein-tyrosine kinases. This review presents a comprehensive overview of novel targets of kinase inhibitors, with a specific focus on cyclin-dependent kinases (CDKs) and epidermal growth factor receptor (EGFR). The majority of the reviewed studies commenced with an assessment of cancer cell lines and concluded with a comprehensive biological evaluation of individual kinase targets. The reviewed articles provide detailed information on the structural features of potent anticancer agents and their specific activity, which refers to their ability to selectively inhibit cancer-promoting kinases including CDKs and EGFR. Additionally, the latest FDA-approved anticancer agents targeting these enzymes were highlighted accordingly.

## 1. Introduction

Cancer remains a leading cause of mortality worldwide, despite significant advancements in the discovery of potential anticancer therapies [1,2,3]. While the FDA approves new cancer drugs annually, the efficacy of current treatments is hampered by multiple drug resistance, and severe side effects. Consequently, there is a pressing need for the development of novel therapeutics with reduced toxicity [4,5,6]. Extensive efforts are underway to identify compounds with improved safety profiles. In pursuit of this goal, researchers are designing and synthesizing novel chemical structures targeting key biological pathways implicated in cancer progression, such as EGFR, CDKs, Ras, and tubulin proteins. These pathways represent primary targets for the development of innovative anticancer agents [7,8].

A protein kinase functions as an enzyme that catalyzes the transfer of the phosphate group of ATP to threonine, serine, and/or tyrosine residues on protein substrates, a process commonly referred to as phosphorylation. Phosphorylation induces a functional modification in the target protein, controlling the signaling pathways. Notably, human genome sequencing has uncovered that approximately 2% of the human genome is dedicated to encoding protein kinases [9]. The human genome encompasses 518 kinases, pivotal enzymes responsible for phosphorylating up to one-third of the proteome [10,11]. Virtually every signal transduction process relies on a phosphotransfer cascade, underscoring the multitude of opportunities kinases offer for therapeutic intervention across various aberrantly regulated biological pathways [12,13]. Beyond cancer, dysregulation of kinase function is implicated in numerous diseases, including immunological, inflammatory, degenerative, metabolic, cardiovascular, and infectious conditions [14,15]. This emphasizes the wide-ranging significance of targeting kinases in the treatment of diverse medical ailments [16,17]. Currently, the FDA has approved 69 therapies that specifically target around 24 protein kinases. These treatments are given for the treatment of neoplastic disorders [18].

**Figure 1:**
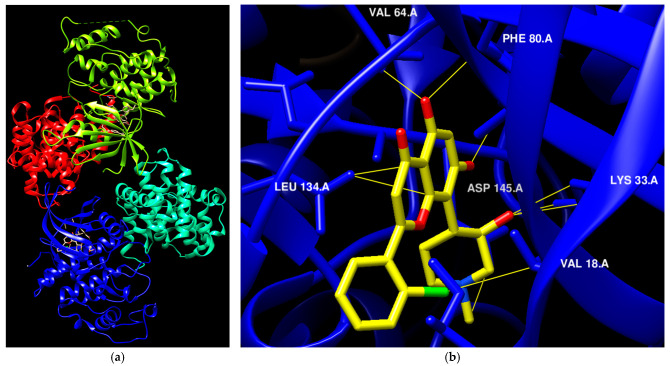

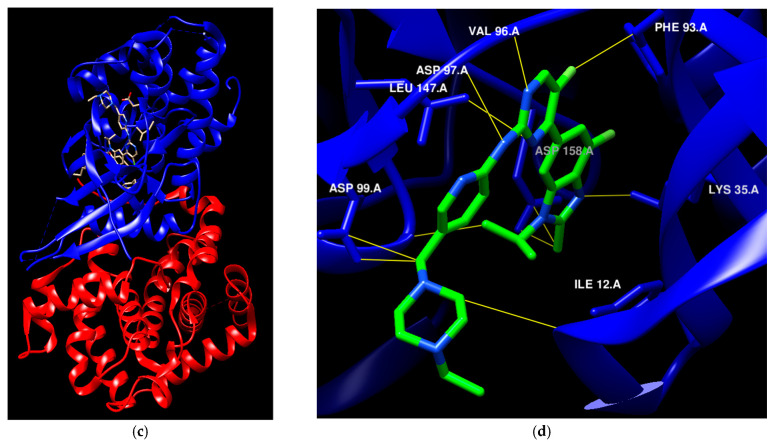
(**a**) The crystal structure of CDK2/CyclinA with Flavopiridol (PDB: 6GUB). (**b**) The binding interactions between Flavopiridol and certain residues in side CDK2. (**c**) The crystal structure of CDK4/CyclinD with abemaciclib (PDB: 7SJ3). (**d**) The binding interactions between abemaciclib and certain residues in side CDK4.

The availability of various protein kinase X-ray crystal structures in the public domain has expedited the process of developing drugs based on structural information [19,20]. The crystal structure with PDB id 6GUB was utilized recently in many works as CDK2/CyclinA complex crystal (Figure 1a) [21,22]. Flavopiridol is considered an inhibitor of multiple cyclin-dependent kinases that causes a cell cycle arrest and apoptosis [23,24]. This agent has been documented to hinder the activity of CDK2 and was subjected to co-crystallization with CDK2 as presented in Figure 1a [25], and the binding mode of this drug was elucidated through the establishment of hydrophilic and hydrophobic interactions with the residues Lys33, Val18, Phe80, Val64, and Leu134 as presented in Figure 1b [21]. A crystal structure assigned the PDB id 7SJ3 has been recently employed in several studies as a CDK4/CyclinD complex crystal with co-crystal abemaciclib (Figure 1c), and the binding mode of this drug was elucidated through the establishment of hydrophilic and hydrophobic interactions with Ile12, Lys35, Phe93, Val96, Asp97, Leu147, and Asp99 residues as presented in Figure 1d [26,27,28]. This ligand was approved by the FDA in 2017 for breast cancer [29]. In addition, commercial ventures have developed and implemented various proprietary structures that are widely utilized in the drug discovery process. There are now around 180 protein kinase inhibitors that can be taken orally that are being tested in clinical trials globally [19,20]. To access an up-to-date and extensive list of these agents, please visit www.icoa.fr/pkidb/ (accessed on 18 July 2024). Approximately 80 medications approved by the FDA specifically target around 24 distinct protein kinases [30].

CDK inhibitors are typically identified by high-throughput, fragment-based screening and virtual methods to facilitate the development of novel anticancer medicines with potent therapeutic properties [31,32]. The progress of the cell cycle and cell division in organisms ranging from yeast to humans is facilitated by the progression of a set of serine-threonine kinases known as CDKs. Several CDKs regulate the cell cycle in mammalians and have long been regarded as crucial for normal cell growth, development, and maintenance of internal stability. The significance of the CDK-cyclin complexes in cell proliferation is emphasized by the discovery that the dysregulation of CDK activity is present in almost all types of human tumors [33,34]. Four FDA-approved antagonists specifically target CDK4/6 for the treatment of breast cancer [35,36]. It is crucial to discuss the processes that regulate the division process of cancer cells in order to hinder tumor growth. The replication of identical daughter cells is a tightly controlled process in healthy cells. Nevertheless, genetic alterations that may take place in cancerous cells can ultimately result in uncontrollable cell growth. To gain a more comprehensive comprehension of the mechanism by which cyclin-dependent kinase inhibitors (CDKIs) work, it is necessary to provide a broad description of the cell cycle and illustrate how cyclins and CDKs can have a substantial impact on the course of the cell cycle. Multiple observations have been conducted to discourage the cell division process in the cancer pathways, leading to the discovery of novel CDKIs. Nevertheless, the intricate nature of cellular control pathways poses significant difficulties in effectively inhibiting the proliferation of tumor cells in a targeted manner [37,38,39].

According to research, eight of the kinase inhibitors that have been approved by the FDA establish covalent connections with the enzymes they target. These inhibitors are classed as TKIs, which stand for targeted covalent inhibitors [40]. The agents mentioned are acalabrutinib, dacomitinib, osimertinib, afatinib, neratinib, zanubrutinib, ritlecitinib, and ibrutinib. These agents have specific targets in various types of cancer, such as blocking Bruton’s tyrosine kinase (BTK) in mantle cell lymphoma, targeting mutant EGFR in NSCLC, or inhibiting ErbB2 in HER2-positive breast cancer, and Waldenström macroglobulinemia. The EGFR and ErbB4, which are closely related members of the ErbB subtype EGFR family, are the protein kinases that most frequently exhibit alterations in all types of malignancies [41].

FDA-approved TKIs targeting EGFR, such as first- and second-generation drugs like erlotinib and afatinib, often face resistance within 8 to 14 months due to mutations like T790M [42,43]. Third-generation TKIs, such as osimertinib, were developed to address this, but resistance still develops through mechanisms like the C797S mutation and activation of alternative pathways (e.g., MET amplification, HER2 overexpression). Fourth-generation TKIs are in development to overcome these issues, but challenges remain in achieving selectivity and reducing side effects. Recent clinical evidence highlights the need for combination therapies and more advanced inhibitors to address both primary mutations and resistance mechanisms [44,45,46].

**Figure 2:**
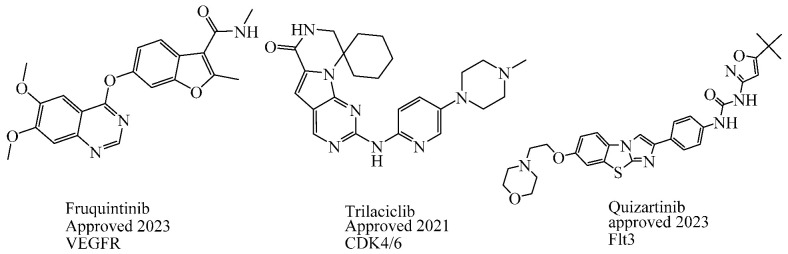
The molecular compositions of various newly authorized anticancer drugs that specifically act on kinase families such as VEGFR, CDKs, and Flt3.

In the past two decades, the FDA has approved more than 10 anticancer drugs associated with EGFR. Additionally, in 2023, fruquintinib, a dimethoxyquinazoline derivative (Figure 2), was sanctioned by the FDA as a novel therapeutic agent aimed at targeting the vascular endothelial growth factor receptor (VEGFR) for managing metastatic colorectal cancer [47]. Over the past decade, the FDA has approved four drugs targeting CDK4/6. Trilaciclib, a derivative of piperazine-pyridine-amino-spiro (Figure 2), is the latest medicine to be licensed in this family. It is recognized for its ability to protect the bone marrow and its potential to effectively combat the proliferation of cancerous cells and provide safety advantages when used alongside cancer therapy [48]. Other drugs that specifically target the receptor protein-tyrosine kinase have recently been recognized by the FDA in 2023, like Quizartinib (Figure 2), which belongs to the benzothiazole phenyl-urea derivative scaffold and selectively targets the Flt3 protein. As a result, this drug has been used to treat acute myelogenous leukemia and has shown excellent observations [49,50].

This study provides a comprehensive analysis of the latest advancements in anticancer drugs or substances that primarily focus on protein kinase enzymes. The information offered contains comprehensive details regarding the chemical structures, IUPAC nomenclature, mechanisms of action, structure-activity relationships (SAR), targeted cancer cell lines, and developmental statuses of highly potent inhibitors. The focus is specifically on CDKIs and EGFR inhibitors. Moreover, an updated inventory of approved drugs within this classification has been compiled. Notably, a significant portion of recently discovered compounds fall under the category of kinase inhibitors. For a deeper understanding, readers are encouraged to explore this review article, which offers a comprehensive analysis of the kinase inhibitors’ landscape alongside their respective references.

## 2. Cyclin-Dependent Kinases

The CDK family is pivotal in regulating essential processes such as cell cycle progression, transcription, and splicing. Dysregulation at any of these stages can induce programmed cell death, known as apoptosis. Failure to correct these dysfunctions may lead to the development of various diseases, prominently including cancer and neurodegenerative disorders [51]. CDKs are well-established as key regulators of cell proliferation. Consequently, current research efforts are primarily directed toward elucidating the intricate connections between CDK/cyclin complexes and signal transduction pathways that govern cell growth, differentiation, and apoptosis. This emphasis seeks to reveal innovative prospects for the diagnosis and treatment of cancer and other conditions linked to aberrant cell growth [52,53]. Therefore, it is crucial to thoroughly examine the mechanisms of CDK inhibitors and explore their clinical applications, particularly in light of recent updates in the literature. This endeavor is essential for gaining deeper insights into CDK inhibitors, their various classes, and their pivotal role in cancer treatment [54].

### 2.1. FDA-Approved CDK Inhibitors

In the past decade, the FDA has approved four CDK inhibitors belonging to the CDK4/6 subtype, as listed in Table 1. These inhibitors are categorized as protein-serine/threonine kinase inhibitors. Notably, these four drugs share a common core structure characterized by a piperazin-pyridin-pyrimidin-amino scaffold. They exhibit similar features, including binding to the inactive kinase conformation. Additionally, they establish hydrogen bonds between the 3N-pyridine moiety and the NH group of HIS100, as well as between the carbonyl (CO) group of Val101 and the exocyclic NH group of the side chain [55].

CDKs do not just control the cell cycle; they also have other roles. For example, CDK1 is involved in phosphorylation of Bcl-2 family proteins, and CDK4 is involved in glucose metabolism [60,61]. CDKs remain inactive unless they are bound to cyclins. When they are not phosphorylated, their access to the active site is impeded by a flexible structure referred to as the activation loop or T-loop. [62]. When cyclins bind to CDKs, significant structural changes occur. This rearranges the ATP-binding pocket, preparing it for the phosphotransferase reaction. The most noticeable change happens in the T-loop, which becomes almost flat at the entrance of the cleft. This alteration removes the barrier from the catalytic cleft, allowing the substrate to bind with the protein [63,64]. CDKs and cyclins are frequently disrupted in different cancers, leading to the unchecked growth of cancer cells. Recent research also indicates a connection between cancer and irregularities in transcription factors. Table 2 outlines the varied biological functions of the main CDKs and their cyclin partners, highlighting their significant involvement in various types of cancer.

### 2.2. Cyclin-Dependent Kinase 2

Anomalous activation of CDK2 has been recognized as a primary mechanism of resistance to CDK4/6 inhibition in hormone-receptor-positive (HR+) breast cancer. Furthermore, there is reliable preclinical evidence that highlights the essential function of this medication in promoting the survival of cancer types characterized by overexpression [74]. Several molecular docking studies have been conducted to evaluate the binding modes within the CDK2 binding pocket. Borik et al. designed novel heterocyclic derivatives based on curcumin for cytotoxic activity. Among these, the most potent derivative demonstrated the best virtual effect on MCF-7 cells, with molecular docking revealing strong binding energy and key hydrogen/hydrophobic interactions with the CDK2 binding pocket [75]. Similarly, Riyadi et al. synthesized thiazolo-indol derivatives, with two compounds emerging as the most active CDK2 inhibitors. Docking simulations showed interactions with Lys33, Glu81, and Leu83 in CDK2, mimicking ATP binding [76].

#### 2.2.1. Pyrazole, Pyrimidine, and Related Derivatives as CDK2 Inhibitors

Pyrazole compounds demonstrate a diverse array of pharmacological activity, encompassing anti-inflammatory, antipyretic, and analgesic properties [77,78], and especially as anticancer effects [78,79,80]. Srinivasulu et al. synthesized a derivative of disubstituted pyrazolo-pyrimidine analogs and assessed their antitumor activities. The derivatives were tested for their effects on CDK2/cyclin E besides Abl kinases; additionally, their antiproliferative effects against MCF-7 and K-562 cancer lines were investigated. Among these derivatives, St.1 (Table 3) demonstrated the highest activity against the mentioned cell lines with potent inhibitory activity against CDK2/cyclin E. Importantly, cytotoxicity studies on normal cell lines indicated that all compounds were non-toxic to normal cells. SAR analysis revealed that the anticancer activity of these compounds was influenced by substituents at positions 4 and 6 on the pyrazolo-pyrimidine scaffold. Compounds with substitutions at these positions showed enhanced activity compared to those with only 6th position substitutions. Additionally, the incorporation of the benzofuran group at the 4th position exhibited superior activity compared to furan and thiophene substitutions. Surprisingly, compounds substituted at the 6th position also showed improved activity compared to phenyl-carbamoyl acetamide substitution [81].

Samar et al. synthesized a series of derivatives and evaluated their CDK2 inhibitory activity. Among the synthesized compounds, St.2 and St.3 (Table 3) exhibited the most potent CDK2 inhibitory activity. St.2 demonstrated 1.4 and 2.3-fold inhibition of MOLT-4 and HL-60 cells, respectively, compared to dinaciclib. Pharmacokinetic analysis revealed that both compounds have good oral bioavailability and high gastrointestinal absorption but cannot penetrate the blood–brain barrier. The synthesis of new CDK2 inhibitors bearing the pyrazol, pyrimidine core represents a significant advancement. SAR analyses indicated that substitution patterns of halogens on the phenyl rings significantly influence cytotoxic activity. The bromo at the *para* position of the “A” phenyl ring enhances anti-tumor activity, while the chloro substituents in the *ortho* or *meta* positions at the “B” phenyl ring are more effective than in the *para* position for both cytotoxic and CDK2 inhibitory activities [82].

Basma et al. synthesized a series of pyrazolo-pyridines and investigated their anticancer effects on MCF-7, Hela, and HCT116 cancer cell lines. St.4 (Table 3) exhibited the highest anticancer activity against HeLa among the derivatives, comparable to that of the standard drug doxorubicin. Similarly, compound St.5 demonstrated significant cytotoxicity against MCF-7 and HCT116 cell lines. Both compounds induced cell cycle arrest and apoptosis in the tested cancer cell lines, with St.4 showing S phase arrest in Hela cells and St.5 inducing G2/M phase arrest in MCF-7 and S phase arrest in HCT116 cells. Furthermore, St.4 and St.5 demonstrated inhibitory activity against CDK2 and CDK9. Molecular docking studies indicated that both compounds fit well in the active sites of both CDK2 and CDK9, suggesting a mechanism for their action against cancer cells. The electronic properties of phenyl substituents at the 4th position of the pyridine ring significantly influenced the anticancer activity of these compounds. Substitution variations in compound series resulted in varied effectiveness against Hela cells, with compound St.4 showing superior activity [83]. In another work, a series of pyrazolo-pyrdine derivatives were synthesized, and among the synthesized compounds, St.6 and St.7 (Table 3) observed potent activities against CKD2/Cyclin A with 96% inhibition on this enzyme for both compounds at 10 µM concentration [84].

Fanta et al. designed a series of pyrazol-pyrimidine-amine as potential anticancer agents targeting CDK2. Among the investigated derivatives, St.8 (Table 3) showed the best activities against various cancer cell lines besides potent activities on CDK2, as well as this compound, which arrests the cell cycle at S and G2/M phases and induces apoptosis [85]. Cheng et al. synthesized a series of pyrazole carboxamides as CDK2 inhibitors, and they present a collection of novel inhibitors that target histone deacetylase (HDAC). Among the synthesized derivatives, St.9 and St.10 (Table 3) exhibited potent antiproliferative effects against five solid cancer cell lines. Moreover, these compounds demonstrated excellent inhibitory activities against HDAC2 with IC_50_ values of 0.25 and 0.24 nM, respectively, and both of these compounds significantly suppressed the migration of A375 and H460 cells. Further investigations revealed that these compounds induced cell cycle arrest in the G2/M phase and promoted apoptosis in A375, HCT116, H460, and HeLa cells, which was associated with elevated intracellular reactive oxygen species (ROS) levels. The presence of an electron-donating methoxyl group resulted in diminished activity compared to compounds with electron-withdrawing groups; substitution with fluorine or chlorine moieties at the *ortho* positions of the phenylamide ring enhanced activity relative to compounds with hydrogen atoms. Notably, compounds St.9 and St.10, featuring two chlorine atoms at *ortho* positions, demonstrated excellent antiproliferative activities. The pyrazole-3-carboxamide group of these two compounds was observed to form hydrogen bonds with the backbone residues Glu81 and Leu83 in the hinge area of CDK2, which are essential for CDK2 inhibition. Importantly, compound St.9 exhibited favorable pharmacokinetic properties, including an intraperitoneal bioavailability of 63.6% in ICR mice, and demonstrated potent in vivo antitumor efficacy in the HCT116 xenograft model [86].

In a recent study by Shaker et al., a series of pyrazole-triaryl derivatives were designed, synthesized, and investigated as potential anticancer agents targeting CDK2 and cyclooxygenase-2 (COX-2) enzymes. All of the evaluated derivatives underwent screening against three cancer cell lines [87]. The COX-2 enzyme is consistently produced in excessive amounts in different types of cancer in humans due to signaling pathways involving protein kinase C and RAS. It has been observed that selective COX-2 inhibitors can affect the checkpoints in the cell cycle by reducing the levels of cyclin D1 and cyclin E. Therefore, CDK2 and COX-2 are potential targets for cancer treatment [87,88]. Among the synthesized series, St.11 (Table 3) was the most active compound and found to induce apoptosis of HepG2 cells by regulating the G1 phase of the cell cycle. Furthermore, it markedly reduced the levels of anti-apoptotic Bcl-2 expression and increased the levels of pro-apoptotic Bax expression, thus demonstrating the cells’ vulnerability to apoptosis. Molecular modeling studies have revealed that the chemicals’ anticancer effect is achieved via inhibiting the CDK2 and COX-2 enzymes. Hence, diaryl pyrazole compounds with a methylsulfonyl group could potentially provide a straightforward means to develop highly effective inhibitors of CDK2 and COX-2, which possess notable anticancer properties [87].

**Table 3:** The structures and the IC_50_ values against a panel of cancer cell lines and CDK2 for the most active agents, which contain pyrazole, pyrimidine, and pyridine scaffolds.

Code	Structures	Evaluated Cancer Cell Lines		CDK2	Ref.
		Cell lines	IC_50_/IG_50_	IC_50_	
St.1	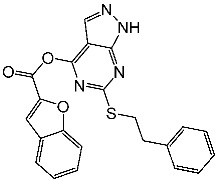	K-562 MCF-7	19.8 µM 18.9 µM	6.8 µM	[81]
St.2	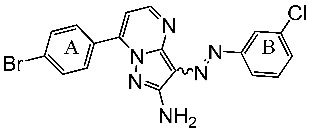	MOLT-4 HL-60	0.93 µM 0.80 µM	22 nM	[82]
St.3	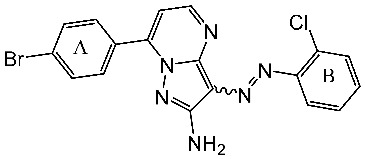	MOLT-4 HL-60	1.28 µM 0.92 µM	24 nM	
St.4	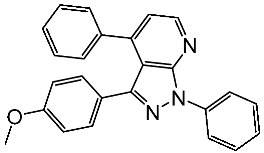	HeLa	2.59 μM	1.63 μM	[83]
St.5	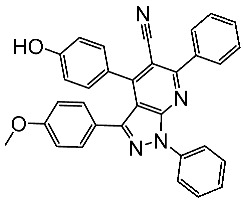	HCT116 MCF-7	4.66 μM 1.98 μM	0.46 μM	
St.6	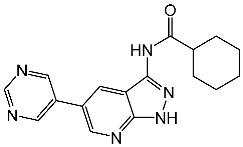	-	-	0.36 μM	[84]
St.7	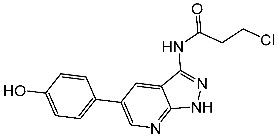	-	-	0.66 μM	
St.8	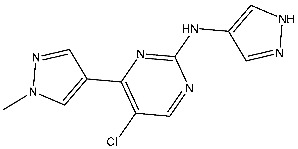	A2780 MCF-7	0.158 μM 0.342 μM	CDK2/E Ki = 0.005 μM	[85]
St.9	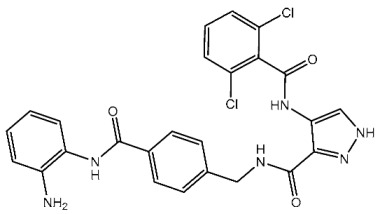	HCT116 A375 HeLa H460 SMMC77	0.71 μM 1.20 μM 1.83 μM 4.19 μM 7.76 μM	0.30 nM	[86]
St.10	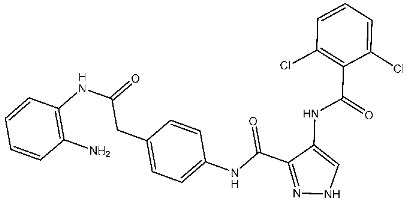	HCT116 A375 HeLa H460 SMMC77	1.45 μM 1.60 μM 3.15 μM 2.63 μM 5.22 μM	0.56 nM	
St.11	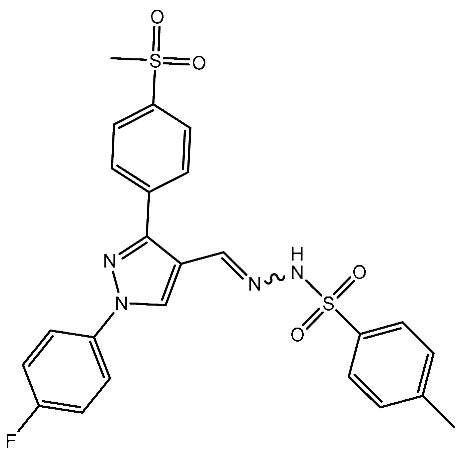	HepG2 HCT116 MCF-7	3.98 μM 8.70 μM 7.85 μM	0.61 μM	[87]
St.12	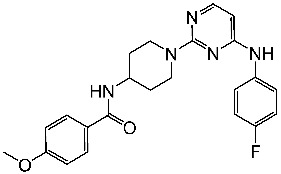	NA	NA	45.4 nM	[89]
St.13	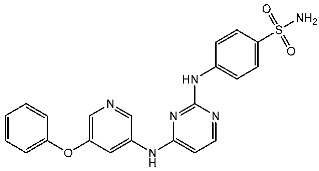	HT-29 MV4-11 MCF-7 HeLa	2.12 μM 0.83 μM 3.12 μM 8.61 μM	64.4 nM	[90]
St.14	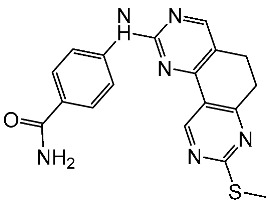	MCF-7 HCT116	1.03 μM 0.9 μM	0.11 μM	[91]
St.15	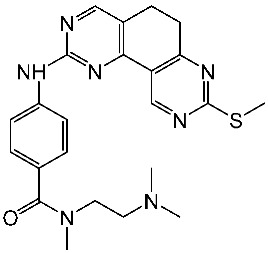	MCF-7 HCT116	1.1 μM 1.4 μM	0.09 μM	
St.16	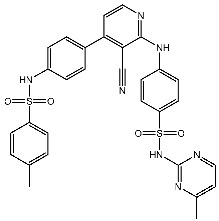	MCF-7	9.10 μM	1.79 μM	[92]
St.17	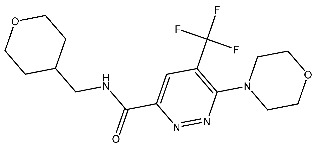	T-47D MDAMB SKOV3	0.43 μM 0.99 μM 15.37 μM	20.1 nM	[93]

Wang et al. designed a series of pyrimidine-piperidine derivatives and tested their activity against CDK2. St.12 (Table 3) emerged as the most active structure, exhibiting potent inhibitory activity against CDK2. Additionally, this compound demonstrated a broad range of anticancer activity against a panel of human breast cancer cell lines by inducing apoptosis and cell cycle arrest, particularly at the G0, G1, and S phases in MDA-MB-468 cells. Further analysis revealed that the presence of a 4-methoxy group in the phenyl amide group was crucial for the activity. Positional variations in substituents indicated that a 4-fluoro group in the amino-phenyl ring conferred the highest activity, while substitution with 2-methyl or 4-methyl led to a decrease in activity. This structure was found to form important interactions with key residues in the ATP-binding site of CDK2, including hydrogen bonding with Leu83 residue and anion interaction with Asp86 residue. These interactions contributed to the binding affinity with CDK2, emphasizing its capacity as a therapeutic agent for the treatment of breast cancer [89].

Zeng and colleagues designed a new series of pyrimidine-amino-pyridine by modifying the similar structures containing the piperidine-amino-pyrimidine; among the new series St.13 (Table 3) showed promising results against different cancer cell lines and CDK2, in comparison with the Palbociclib positive control. Molecular docking studies were conducted to investigate the interactions between this compound binding pocket of CDK2. This compound attaches to the ATP-binding site in a U-shaped conformer, exhibiting a binding mechanism comparable to that of AZD5438, the ligand of the co-crystal [90].

Hu and co-workers designed and synthesized a series of pyrimido-quinazoline derivatives, and observed their potential as CDK2 inhibitors. Compounds St.14 and St.15 (Table 3) demonstrated significant activities against the CDK2 enzyme and exhibited strong antiproliferative activities against MCF-7 and HCT116 cancer cell lines. The two substituted with phenylamine among these derivatives showed the best overall anticancer activity compared to other derivatives. Furthermore, the tricyclic scaffold of pyrimido-quinazoline, identified as an inhibitor of various cell cycle kinases, including CDK2, interacts with Leu83 residue at the ATP site. The pyrimidine portion of this scaffold forms crucial hydrogen bonds with the CDK2 hinge region (Leu83), essential for inhibitory activity [91].

Ghorab et al. conducted the synthesis of amino pyridine derivatives which were designed as potential anticancer agents. These synthesized compounds underwent biological evaluation for cytotoxic activity against MCF-7 cell lines. Within this series, compound St.16 (Table 3) exhibited notable activities against both the MCF-7 and CDK2 enzymes. Additionally, the potential of γ-radiation to enhance the cytotoxic activity of this compound was investigated, demonstrating a significant increase in cell killing effect at lower concentrations post-irradiation. Docking studies were conducted to investigate the potential binding modes within the active site of the CDK2 enzyme. The residue Leu83 was found to interact with the nitrogen (N) of the pyridine moiety at a distance of 3.02 Å, as well as with the NH group linked to the pyridine at a distance of 2.61 Å. Additionally, the residue Lys 89 exhibited an interaction with the sulfone group (SO_2_) of the sulfonamide moiety at a distance of 3.09 Å, while Asp86 interacted with the SO_2_ group of the sulfonamide at a distance of 2.89 Å. Moreover, Tyr15 was observed to interact with the SO_2_ group of the tolyl sulfonamide moiety at a distance of 3.04 Å [92].

Sabt et al. synthesized a series of pyridazine derivatives with anticancer activities aimed at targeting the CDK2 enzyme, utilizing the 3,6-disubstituted pyridazine scaffold to enhance the therapeutic arsenal with efficient and safe anticancer CDK inhibitors. The synthesized compounds underwent evaluation for in vitro CDK2 inhibitory activity. These compounds demonstrated potent anti-proliferative effects against T-47D and MDA-MB-231 cell lines. Notably, among the synthesized compounds, St.17 (Table 3) emerged as the most active compound. This compound showed various binding interactions inside the pocket of the CDK2 active site, including a hydrophobic interaction with non-polar residues and hydrogen bonding with the other residues. Moreover, this compound induced cell cycle arrest at the G2/M phase and triggered apoptosis and necrosis in both T-47D breast cancer and MDA-MB-231 cell lines. [93].

#### 2.2.2. Thiazole, Thiouracil, and Related Derivatives as CDK2 Inhibitors

Thiazole derivatives are recognized as agents with diverse biological activities, encompassing anticancer properties [94,95], neuroprotective effects [95], and anti-inflammatory capabilities [96,97]. El-Naggar et al. synthesized a novel series of thiazole-hydrazine derivatives with potential as CDK2 inhibitors. All synthesized compounds were tested for their antiproliferative activities against four cancer cell lines. Among the synthesized series compound, St.18 (Table 4) was the most active derivative on CDK2, exhibiting the highest potency, being two-fold more potent than the roscovitine positive control against this target. Moreover, this compound was observed to arrest the cell cycle at the G2/M phase of HepG2 and have apoptotic effects on the same cell lines. Docking studies indicated that these derivatives are well accommodated within the binding pocket of the CDK2, engaging in various binding interactions with seven amino acid residues. This structure showed the best docking values (−18.63 kcal/mol) and exhibited significant activities against the CDK2, nearly matching the score of roscovitine (−17.03 kcal/mol). A strong binding interaction involving hydrogen bonds between the N atoms of thiazole and hydrazinyl of this compound and the Lys89 amino acid was observed. Additionally, the CH_3_ group, which is linked to thiazole, may potentially form binding interactions within the active site, such as hydrophobic interactions with His84 [98].

A series of derivatives, based on dichlorophenoxymethyl and featuring various nitrogenous heterocyclic rings were synthesized and evaluated on cancer cell lines and the CDK2 enzyme. Among this series, thioxonaphtho-oxazine containing compound St.19 and benzothiazole containing compound St.20 (Table 4) were the most potent compounds that were synthesized via the hybridization concept. The derivatives show promising results against CDK-2 in comparison with roscovitine as a positive control. To elucidate the potential binding interactions of the most active compounds in this series, molecular docking simulations were conducted inside the pocket of the CDK-2. St.19 showed strong binding interactions, including hydrogen bond interactions with two oxygens of the sulfonic moiety (with distances ranging from 2.63 to 2.86 Å). Furthermore, hydrogen bond donor interactions were observed between the side chain of Lys89 and the oxygens of the 2,4-dichlorophenoxy moiety in this compound. Similar binding modes of derivatives St.20 were observed inside the ATP-binding pocket of CDK-2. The backbone of Leu83 amino acid is bound via hydrogen-binding interactions with N and NH groups of benzo-thiazole and amide groups. The excellent inhibitory activity observed can be attributed to these specific interactions, highlighting the superior activity of St.19 due to the additional hydrogen bonding, which is crucial for a more precise fitting within the active site of CDK-2 [99].

Hendawy and colleagues designed a series of thiazolidinone analogs and tested their anticancer activities by targeting CDK2 and EGFR, as well as the apoptotic effect observed in three caspases (3, 8, and 9). St.21 and St.22 (Table 4) demonstrated strong inhibitory activity against CDK2 and EGFR. These compounds also increased the activity of caspases 3, 8, and 9, as well as cytochrome C levels in the breast cancer cell lines. The SAR analysis indicated that compounds featuring the 2,4-dinitrophenyl-hydrazono-thiazolidine-4-one moiety exhibited higher antiproliferative activity compared to the other derivatives. Furthermore, the substitution of p-tolyl (St.22) was better than cyclohexyl (St.21) or other substitutions, and p-tolyl showed better antiproliferative, CDK2 inhibitory, and EGFR inhibitory activities. Molecular docking simulations revealed that the test compounds were stabilized within the active site cavity of the target proteins, forming hydrogen bonds and π-hydrophobic binding interactions with some amino acids [100]. In another work on this series scaffold (thiazolidinone), two compounds were synthesized by a rapid method called grindings without using any solvent, and these two compounds were evaluated virtually, and they showed a promising virtual binding interaction with the CDK2 binding pocket [101].

In a recent study, Manda et al. created a series of thiazolidinone-coumarin derivatives as potential anticancer compounds that target the CDK2 enzyme. These novel compounds were synthesized by using the hybridization concept, and they were evaluated for their in vitro activities against both MCF-7 and CDK2. The most active agent was St.23 (Table 4) and showed promising activities against these targets. Molecular docking simulations demonstrated the engagement of the St.23 compound with the CDK2 binding pocket via hydrogen bonding interactions. The carbonyl (C=O) group of the benzylidene ring forms a hydrogen bond with the Leu281, and another binding interaction occurs through the methoxy group of the phenyl ring Glu8 [102].

Fatahala et al. innovated a series of thiouracil-5-sulfonamide derivatives as potential anticancer agents targeting the CDK2 enzyme. The anticancer effects of these derivatives were assessed against a panel of cancer cell lines. The most potent compound was St.24 (Table 4), which arrests the cell cycle at G1/S, S, and G2/M phases in A-2780, HT-29, MCF-7, and HepG2 cells, respectively. Moreover, this compound showed an apoptosis effect in all utilized cancer cells. In terms of SAR analysis, derivatives without the SO_2_NH group showed inactivity against the mentioned cancer cell lines, while the St.24 compound, which has SO_2_NH and contains 2,3-dichlorophenyl, demonstrated the highest potency. These observations suggest the critical role of the sulfonamide group in cytotoxic activity. However, further modification of this group by converting it to SO_2_NHNH led to a reduction in potency. Molecular modeling revealed the binding of this compound by forming hydrogen bonds with amino acids (Gln131, Lys33, and Lys129) [103].

**Table 4:** The structures and the IC_50_ values against a panel of cancer cell lines and CDK2 for the most active agents which contain Thiazole, and Thiouracil scaffolds.

Code	Structure	Evaluated Cancer Cell Lines		CDK2	Ref.
		Cell Lines	IC_50_ or IG_50_	IC_50_	
St.18	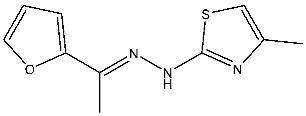	HepG2 MCF-7 HCT116 WI-38	8.49 µM 17.09 µM 22.50 µM 73.45 µM	0.35 µM	[98]
St.19	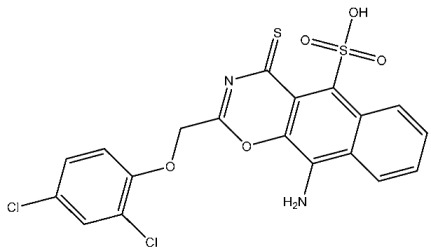	HCT116 MCF-7	3.78 µM 6.41 µM	0.21 µM	[99]
St.20	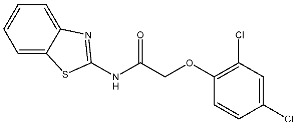	HCT116 MCF-7	5.91 µM 7.39 µM	0.70 µM	
St.21	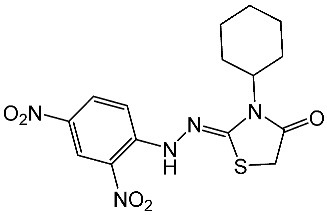	Panc-1 MCF-7 HT-29 A-549	0.80 µM 0.65 µM 0.90 µM 0.95 µM	18 nM	[100]
St.22	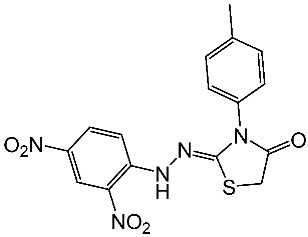	Panc-1 MCF-7 HT-29 A-549	0.70 µM 0.60 µM 0.80 µM 0.80 µM	14 nM	
St.23	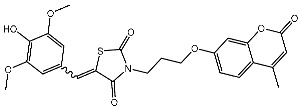	MCF-7	12.15 µg/mL	7.5 µg/mL	[102]
St.24	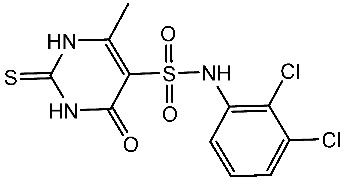	A2780 HT29 MCF-7 HepG2	2.52 µM 1.75 µM 1.67 µM 2.02 µM	0.41 µM	[103]
St.25	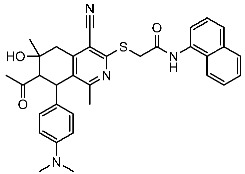	MCF-7 A459	0.21 µM 0.15 µM	149 nM	[104]

Sayed et al. developed and created a new series of tetrahydroisoquinolines. Within this collection, St.25 (Table 4) stands out as it contains thio-naphthalen-acetamide connected to cyano tetrahydroisoquinolines that have shown a significant efficacy on several cell lines in comparison to the positive control drug doxorubicin. This compound induces cell cycle arrest specifically at the G2/M phase. Based on the docking analysis, this compound exhibited a higher binding affinity with a binding score of −10.3 kcal/mol to CDK2 compared to the standard STU299 value (−11.5 kcal/mol). The examination of the binding interactions revealed that this compound established hydrogen and hydrophobic interactions bonding, with important amino acid residues in the CDK2 binding region, such as Glu12, Val18, Lys33, and Leu134 [104].

### 2.3. Cyclin Dependent Kinase 4/6

In recent years, four CDK4/6 inhibitors have been approved by the FDA and are highlighted in Table 1. These medicines have been observed to have low permeability through the blood–brain barrier (BBB). Hence, there is a constant need for the advancement of CDK4/6 inhibitors that have been clinically authorized for the treatment of brain malignancies such as glioblastoma multiforme. Due to the disadvantages of these inhibitors in patient treatment resulting from either intrinsic or acquired resistance. Therefore, it is essential to prioritize the identification of many strategies to overcome this challenge. An elucidation of the many processes by which resistance to CDK4/6 inhibitors arises could assist in the development of innovative therapeutic approaches to enhance patient outcomes [35,105,106].

#### 2.3.1. Pyrimidine Derivatives as CDK4/6 Inhibitors

Over the past decade, numerous studies have focused on the synthesis and investigation of the pyrimidine series as the primary framework, intending to target the CDK4/6 [107,108,109,110]. The pyrido-pyrimidine scaffold showed promising activities as an anticancer agent by targeting CDK4/6 and other kinases [110,111,112]. Abbas and his co-workers developed a series of pyrido-pyrimidines as CDK4/6 inhibitors. Among this series, St.26 and St.27 (Table 5) showed significant activities against a panel of cancer cell lines and CDK6 enzymes. Additionally, these two compounds induced apoptosis in PC-3 and MCF-7 via the activation of caspase 3 in PC-3 cell lines, and bax and p53, as well as down regulation of Bcl2. According to the SAR analysis, the size of the atom and its lipophilicity may impact the activity, as demonstrated by the increased activity of chloro derivatives like St.26 and St.27 better than compounds with a fluoro atom. St.26 was found to dock within the binding pocket of CDK6 well by forming hydrogen bonds with Glu99 and Val101 amino acids, in addition to engaging in hydrophobic interactions with other amino acid residues. This direct binding mechanism suggests inhibition of CDK6 activity, alongside activation of the intrinsic apoptotic pathway [113]. In another work, Al-Attraqchi et al. synthesized a series of pyrido-pyrimidines as CDK4 inhibitors; the most potent compound against CDK4 was St.28 (Table 5). According to the molecular docking analysis, the amine group makes a hydrogen bonding interaction with Val96, and another hydrogen bonding interaction is established between the side chain of His95 and the nitrogen atom in the ring. The π-ring system is sustained within the binding pocket through several hydrophobic and π-alkyl interactions with certain hydrophobic residues, namely Aal33, Leu147, Val72, and Ala157 [114]. Fang and his coworkers designed and synthesized a series of pyridin-amino-pyrido-pyrimidine derivatives as CDK4/6 inhibitors based on the chemical structure of the piperazine moiety of the anticancer agent palbociclib. These compounds showed significant anticancer activities against several cancer cell lines, including HepG2, A549, MDA-MB-231, and MCF-7. Among this series, compound St.29 (Table 5) showed a potent inhibitory effect against MDA-MB-231 and MCF-7 breast cancer cell lines with low IC_50_ values, as well as a selective inhibitory effect against CDK4/6. This compound also arrests the cell cycle at the G0/G1 phase and induces apoptosis [115].

Shi et al. conducted research on a novel series of pyrrolo-pyrimidines, and this class of compounds was synthesized and evaluated for its impact on pancreatic cancer cells, focusing on in vitro studies and activity against CDK4/6. Among the synthesized derivatives, a series of 6-anilino carbonyl-substituted pyrrolo[2,3-d]pyrimidine derivatives demonstrated enhanced potency against various cell lines, specifically St.30 (Table 5) was the most potent agent. Further investigation revealed that this compound exhibited potential for combination therapy with mTOR inhibitors in pancreatic cancer treatment. This was achieved by introducing a sulfonamide group on the C2-substituent of pyrrolo[2,3-d]pyrimidine, which affected CDK activity. CDK is pivotal in regulating cell cycle progression from the G1 to S phase, making it a promising target for cancer therapy [116]. Another work by Sroor and his co-workers on the pyrrolo-pyrimidines family was conducted to evaluate their anticancer activities. This new set of pyrrolo[2,3-d]pyrimidine derivatives was created, synthesized, and examined for their antiproliferative activities against several cancer cell lines. Among this series, St.31 (Table 5) showed promising activities as an anticancer agent, as well as a down regulation observed by this compound on CDK4 and BcL2, and this compound arrests the cell cycle at the G1/S phase in MCF-7 [117].

Guo and colleagues synthesized a series of derivatives falling under the same series (pyrrolo-pyrimidines). The synthesized compounds underwent evaluation for their in vitro inhibitory activity against CDK4/6, as well as their anti-proliferative effects on T47D and A549 cancer cell lines. Among these derivatives, St.32 (Table 5) was the most potent against CDK4, demonstrating dual inhibition against both kinase enzymes 4/6. The selective CDK4 inhibitor St.32 displayed antitumor activity by arresting the cell cycle at the G1 phase. This compound is tightly bound to the ATP-binding site the like ribociclib positive control and forms hydrogen bond interactions through amino-pyrimidine and carboxamide groups with His95, Val96, and Asp158 amino acids [118].

Divya et al. designed a series of thieno-pyrimidin-hydrazones as CDK4 inhibitors. A total of 59 derivatives were designed and subjected to evaluation for their inhibitory activity against CDK4/D. According to their findings, St.33 (Table 5) was the most potent compound, which induced cell cycle arrest at the G1 to S phase transition. In the docking study, this compound can form hydrogen bonding interactions with Val96 and Asp99 amino acids within the pocket of CDK4, via the NH of the hydrazone moiety acting as a hydrogen bond donor to the carbonyl group of Val96. Additionally, the nitrogen atom at the 3rd position in the Thieno[2,3-d]pyrimidine moiety acted as a hydrogen bond acceptor [119].

#### 2.3.2. Miscellaneous Derivatives CDK4/6 Inhibitors

Li and his coworkers synthesized a series of pteridin-7(8H)-one derivatives as significant CDK4/6 inhibitors. Among this series, the most potent compound St.34 (Table 6) showed significant anticancer activities against a panel of cancer cell lines, including HCT116, MDA-MB-231, HeLa, and HT-29 cells, with low IC_50_ values, in comparison with the anticancer drug Palbociclib. This compound showed promising activities toward both CDK4 and CDK6 with cyclin D3. As well as, it showed cell cycle arrest at the G2/M phase and caused apoptosis in HeLa cells via a concentration-dependent manner. The sulfamoyl substituent was essential for the activities, and a bulky lipophilic group like cyclopentyl was better than small or hydrophilic substituents. Regarding the molecular docking analysis, compound St.34 observed a similar binding interaction mode like positive control Palbociclib and was located very well in the ATP-binding site of CDK6 with a powerful hydrogen bond interaction via the carbonyl oxygen atom of the pteridin skeleton with the nitrogen of the Asp163 amino acid; moreover, the sulfamoyl moiety interacts with the amino acids Asp102, Thr107, and Gln103 through hydrogen bond interactions [120].

Ali et al. synthesized and evaluated a series of 2-phenyl benzimidazole derivatives as CDK6 inhibitors, among the synthesized series compound St.35 (Table 6), observed promising results against a panel of cancer cell lines with a very high growth inhibition percentage against HL-60, NCI-H522, HCT-15, PC-3, and MCF-7 with an inhibitory percentage higher than 71%, and this compound showed dual inhibition of CDK-6 and Aurora A kinases, as well as cell cycle arrest, was observed at the G1 phase and induced total apoptosis on cancer cell line HCT-15 cells by 45.63%. The molecular docking studies showed a promising score for this compound, which was -9.1 kcal/mol through various hydrophobic interactions with Val27, Ile19, Asp104, Thr107, Asp102, Ala162, Phe98, Ala41, and Leu152 amino acid residues in the CDK6 binding pocket. Referring to the inhibition of Aurora A and CDK-6 enzymes, it was clear that N atoms containing heterocycles like piperidine were very important for potent activities. Additionally, the substitution at position 5th of the benzimidazole ring was essential for the activities, especially with an electron-withdrawing group (EWG) like the nitro group of this compound in comparison with electron-donating groups [121].

Yousuf et al. investigated dietary phytochemicals including rosmarinic acid, ferulic acid, capsaicin, limonene, tocopherol, ursolic acid, caffeic acid, and ellagic acid for their effect on inhibiting CDK6. Among these dietary phytochemicals, ellagic acid (St.36, Table 6) was best located inside the binding pocket of CDK6, this compound inhibited the CDK6 with an IC_50_ value of 3.053 µM, as well as decreased the colonization of cancer cells and induced apoptosis [122].

### 2.4. Cyclin-Dependent Kinase 9

CDK9 is a key controller of transcription that regulates the process of transcription elongation by adding phosphate groups to RNA polymerase II. Temporary suppression of CDK9 leads to the reduction in short-lived transcripts, ultimately inducing apoptosis in cancer cells [123,124]. The overexpression of genes that control tumor cell proliferation, survival, cell cycle regulation, DNA damage repair, and metastasis has been associated with the excessive activity of CDK9 in cancer [125,126]. Multiple CDK9 inhibitors, including fadraciclib, AZD-4573, and CDKI-73, have been created and have shown substantial anti-tumor effects in preclinical research [127]. AZD-4573 is a potent CDK9 inhibitor that specifically reduces the expression of cancer-causing genes such as MCL-1. AZD-4573 is highly effective in treating blood cancers, according to preclinical research [128]. Many novel compounds were designed, synthesized, and targeted CDK9 as anticancer agents [129,130,131]. It was mentioned before that St.6 (Table 3) showed CDK2 inhibitory activities; it also showed significant CDK9 inhibitory activity with an IC_50_ value of 1.8 µM [84].

Ghanem et al. designed, synthesized, and evaluated a series of imidazole-pyridine derivatives as anticancer agents and CDK9 inhibitors. Among the synthesized compounds, St.37 (Table 7) was the most potent derivative, exhibiting superior activity against two cancer cell lines with potent inhibitory effects on CDK9, as well as showed cell cycle arrest at the S phase. The imidazolo-pyridine was essential for activities; the binding affinity of this compound with CDK9 was measured to be −26.72 Kcal/mol. The binding occurred due to the interaction between the amino group, phenyl ring, 1,4-diazepine, carbonitrile group, and pyrimidine moiety and the ATP pocket of the CDK9. The primary amino acid residues implicated in the interaction were Cys106, Ile25, Val33, Asp109, Leu156, Asp167, and Ala46, which was comparable with the native ligand [132].

In another study, a series of chromene-1,2,3-triazole derivatives were synthesized, and the cytotoxicity activity of newly synthesized hybrids was tested against three human cancer cell lines, including MCF-7, MDA-MB 231, and HCT, as well as the in vitro inhibitory potential of all derivatives against CDK9/cyclin T1 was assessed. St.38 (Table 7) which contains a triazole amide linker attached to the terminal chromene was the most potent compound among this series against the cancer cell lines and CDK9 enzyme, as well as this compound showed cell cycle arrest at the G0/G1 phase. It was clear that the selectivity and activity are affected by the size and placement of the substituent on the phenyl ring at the *para* position of the chromene-1,2,3 triazole. The molecular docking analysis was conducted to forecast the binding interactions of the most powerful triazole derivative, St.38, to the CDK9 target’s ATP binding site, three hydrogen binding interactions were observed with Cys106 and Asp167 with distances ranging at 2.84–3.09 Å. By fully occupying the active site, just like the reference control does, it was demonstrated that the design of this compound inhibits the target protein CDK9 very well [133].

In another work, a piperazine derivative was designed and synthesized as a CDK9 inhibitor. This compound, St.39 (Table 7), showed significant activity on several cancer cell lines. Besides potent activities against CDK9 and GSK-3β signal pathways, this compound also effectively suppressed tumor growth in a xenograft mice model with little adverse effects. Molecular docking was conducted to investigate the interaction pattern of St.39 within the active site of CDK9, and based on this model, it was predicted that the 4-carbonyl group has the potential to make a hydrogen bond with Cys106, allowing it to bind to the ATP binding site [134].

Xu et al. designed and synthesized a series of disubstituted pyrimidine as CDK9 inhibitors. Among the synthesized series, compound St.40 (Table 7) showed promising activities and selectivity for CDK9 over CDK2 by 84 folds. This compound induced apoptosis in PANC-1 cancer cell lines, as well as arresting the cell at the G2/M phase of the cell cycle. Molecular docking analysis was conducted to investigate the binding interactions of this structure in CDK9, and it was clear that the *N*-phenylpyrimidin-2-amine forms three hydrogen binding interactions with amino acids Thr29, Asp109, and Asn154 in the binding region of CDK9, as well as that this compound’s anticancer activity was confirmed by in vivo studies in xenograft models [135]. In another study of how pyridine derivatives work as CDK9 inhibitors, Gao et al. synthesized a series of bipyridine derivatives, and among this series, St.41 (Table 7) was the most promising agent with significant activities against various cancer cell lines. This compound also inhibits the cell migration in MDA-MB-231 cancer cell lines and was reported as the first non-metal–organic structure that works as a selective CDK9/Cyclin T1 with in vivo anticancer activities [136].

In a recent work, triazole-pyridine-carbamate derivatives were synthesized and evaluated as CDK9 inhibitors; among this series, St.42 (Table 7) showed potent activities against HCT116 cancer cell lines with significant activities on the CDK9 enzyme. Regarding the molecular docking analysis, the carbamate pyridine and amide moieties formed four hydrogen binding interactions with Cys106 and Asp109, respectively, as well as the benzotriazole moiety formed a π–π binding interactions with Phe103 residue. This structure can trigger apoptosis in the HCT116 cell line by suppressing the phosphorylation of RNA polymerase II at Ser2. This, in turn, leads to the suppression of genes and proteins associated with apoptosis [137].

## 3. EGFR Inhibitors

Tyrosine kinases are enzymes that catalyze the transfer of phosphate groups from ATP to tyrosine residues in cellular proteins, resulting in their phosphorylation [20,138]. The conventional approach for the creation of anticancer drugs involves the utilization of small compounds that inhibit tyrosine kinases. Numerous studies have examined the binding locations and crucial residues in these kinases, intending to develop novel inhibitors. Nevertheless, the process of developing drugs must give priority to selectivity because there are around 30 families of tyrosine kinases [139,140,141]. Receptor tyrosine kinases (RTK) are transmembrane proteins that span the biological membrane and possess extracellular ligand-binding domains (ectodomains) where ligands can attach. Some examples of these proteins are VEGFR, EGFR, platelet-derived growth factor receptor (PDGFR), and fibroblast growth factor receptor (FGFR). Receptor tyrosine kinase has emerged as a primary target for therapeutics in the 21st century [142,143].

The most prevalent medications in targeted therapy are the tyrosine kinase inhibitors (TKIs) that target the EGFR. These TKIs are classified into four generations. First- and second-generation medications result in the development of drug resistance during a period of 8 to 14 months. The primary cause of this resistance is the T790M mutation, which is the most commonly reported mechanism. A new medicine of the third generation has been created to tackle this problem, and a drug of the fourth generation is anticipated to surpass several mechanisms of resistance, including resistance to third-generation drugs [144,145].

### 3.1. FDA-Approved EGFR-Targeting Drugs

The mammalian EGFR family consists of four receptors (EGFR, ErbB2, ErbB3, and ErbB4), which originated via a sequence of gene duplications during the early stages of vertebrate evolution. These receptors share a 40–45% similarity in their genetic makeup [146,147]. Eight EGFR-targeting drugs have been approved by the FDA in the last two decades and are listed in Table 8 as EGFR, ErbB2, and HER2 inhibitors.

### 3.2. New EGFR Inhibitors

Excessive expression of the EGFR leads to aberrant signal transduction and is directly associated with the development of cancer. The majority of EGFR TKIs are inhibitors that compete with ATP. Consequently, the pursuit of targeting the EGFR TK allosteric site has emerged as a very desirable technique for cancer treatment [144,145].

#### 3.2.1. Quinazoline Derivatives as EGFR Inhibitors

The quinazoline core is widely employed in the creation of new EGFR TKIs and as a fundamental component for the synthesis of small compounds with enhanced selectivity [158,159]. Zhang et al. developed and evaluated a series of sulfamoylphenyl-quinazoline derivatives as potential EGFR/CAIX dual inhibitors. These derivatives were evaluated for their cytotoxicity against three cancer cell lines, and the most potent structure was St.43 (Table 9) with superior activities against H1975 cell lines and the EGFRT790M enzyme. This compound demonstrated a noteworthy inhibitory impact on CAIX, similarly to acetazolamide. Additionally, it dramatically suppressed the expression of p-EGFR, as well as its downstream targets p-AKT and p-ERK, in H1975 cells; also, this compound arrested the cell cycle at the G2/M phase [160]. In another work, a new series of quinazoline-based thiazole derivatives were evaluated for their anticancer efficacy in vitro, and compound St.44 (Table 9) was the most potent against different kinds of EGFR mutants, including L858R/T790 M, wild-type, and L858R/T790 M/C797S mutant EGFR kinases. It was clear that derivatives with fluoro, chloro, bromo, trifluoromethyl, or nitro groups were more potent than others with unsubstituted or methyl groups. Compound St.44 is regarded as a good option for future investigation and refinement as EGFR kinase inhibitors with improved anticancer efficacy [161]. In another work with thiazole moiety, a series was developed as EGFR inhibitors, and among this series, St.45 (Table 9) was the most potent with significant nanomolar EGFR inhibitory actions [162]. In a recent other work of the quinazoline derivatives, this series was developed as dual inhibitors of EGFR/c-Met. Among these derivatives, compound St.46 (Table 9) showed remarkable activities on three cancer cell lines and against EGFR L858R and c-Met enzymes, as well as induced apoptosis and cell cycle arrest in A549 cancer cell lines. The in vivo results confirmed the anticancer activities on the same cell lines [163]. In another recent work on the same scaffold (quinazoline), a series was synthesized and showed dual significant activities on EGFR and VEGFR, and compound St.47 (Table 9) was the most promising candidate on both targets [164]. Derivatives of quinoline were developed by Mohassab and his team as EGFR inhibitors. According to the synthesized series, the quinoline, oxime, and methylsulfonyl-phenyl rings were essential for the activities, and the most active compound was St.48 (Table 9). This compound triggered programmed cell death and caused an arrest in the cell cycle, specifically at the S phase. Significantly, upon structural alteration, this compound has the potential to emerge as a very effective inhibitor for both the EGFR and BRAFV600E targets [165].

#### 3.2.2. Pyrimidine Derivatives as EGFR Inhibitors

In recent years, several works on pyrimidine derivatives have been conducted to develop and discover a novel agent as a kinase inhibitor [166,167]. A series of osimertinib derivatives that do not contain the acrylamide group and the pyrimidine group were synthesized as a reversible inhibitor of EGFR. Compounds St.49 and St.50 (Table 10) exhibited significant inhibitory effects against the wild type of EGFR. These two compounds have shown potential and merit additional investigation. Compound St.49 exhibited significant inhibitory efficacy against the L858R/T790 M mutant EGFR kinase as a reversible inhibitor and can cause apoptosis in a dose-dependent manner, arrest the cell cycle at the G1/G0 phase, and limit the motility of A549 and H1975 cancer cell lines, while compound St.50 exhibited exceptional inhibitory activity and selectivity against the mutant EGFR kinase variants L858R/T790 M/C797S [168].

Alanazi and his team developed and evaluated a series of pyrimidine derivatives as multi-kinase inhibitors, including EGFR, VEGFR-2, CDK2, and HER2, besides the evaluation against a panel of cancer cell lines. Among this series, compound St.51 (Table 10) was the most promising candidate with superior activities. This compound showed various binding interactions with different amino acids in the EGFR active site [169]. Elsebaie and her team developed a new series on this scaffold as EGFR inhibitors, and among this series, compound St.52 (Table 10) was the most potent compound on various cancer cell lines with low toxicity on the normal cell lines WI-38, as well as this compound was arresting the cell cycle at G2/m phase in MDA-MB-468 cancer cell lines [170]. In another recent work, a series of pyrimidin-oxazole-carboxamide derivatives were evaluated in silico and in vitro as EGFR kinase inhibitors. These derivatives were tested against a panel of human cancer cell lines, and the most active structure was compound St.53 (Table 10). The featuring of the 3,5-dinitro group on the aryl showed excellent potency on evaluated cancer cell lines. This compound increased the cell population at the G2/M and Sub-G1 cell cycle phases, causing cell cycle arrest at the G2/M phase and induced apoptosis. The molecular docking studies observed that the 3,5-dinitro group on the aryl moiety placed in a hydrophobic pocket exhibited interactions with Leu820, Cys773, Lys721, and Leu764 amino acids; H-bond interactions with the oxazole ring and Lys745; pi–pi interactions with Phe723 Flank with the pyrimidine moiety; and another hydrogen bond interaction with gatekeeper Met793 residue were observed too [171].

#### 3.2.3. Indole Derivatives as EGFR Inhibitors

Several works were conducted to develop indole derivatives as EGFR inhibitors [172,173], Olgen et al. developed a series of indole derivatives and explored their activities on EGFR/SRC kinases. These compounds have the potential to be used as a therapeutic approach for treating EGFR-mutant lung cancer. These derivatives were synthesized via osimetinib’s structure as a basis to address both resistance and adverse effects. Compound St.54 (Table 11) demonstrated the most effectiveness against SRC kinase, and it also caused a notable increase in programmed cell death in cell lines derived from prostate cancer. The results suggest that this compound exhibits dual inhibitory effects on SRC and EGFR kinases while demonstrating modest toxicity toward normal cells [174]. In another work, Al-Wahaibi et al. developed a new series of 5-chloro-indole-2-carboxylate derivatives as EGFR inhibitors. Five compounds in this series exhibited significant activities against EGFR with IC_50_ range values 68–89 nM, and the most potent compound was St.55 (Table 11), as well as this compound showed an 8 times selectivity index toward EGFRT790M protein over EGFRwt [175].

Dubba and Koppula synthesized a new series of indole-isoxazole hybrids as EGFR inhibitors; all compounds were evaluated against two breast cancer cell lines, and among this series, two compounds, St.56 and St.57 (Table 11), showed remarkable inhibitory activities against EGFR [176]. In another recent work, He et al. synthesized a new series of 4-indolyl quinazoline derivatives, and among this series, compound St.58 (Table 11) potently inhibits EGFR and suppresses p-EGFR and p-AKT in lung cancer cell lines. This compound induces apoptosis and arrests the cell cycle at G0/G1 phases [177,178].

**Table 11:** The structures and the IC_50_ values against a panel of cancer cell lines and EGFR subtypes for the most active agents of indole-based derivatives.

Code	Structure	Evaluated Cancer Cell Lines		EGFR or Related	Ref.
		Cell Lines	IC_50_ or Inh.%	IC_50_	
St.54	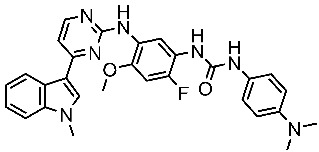	A549 MCF6 PC3	>50%	EGFR = 1.026 µM SRC= 2 nM	[174]
St.55	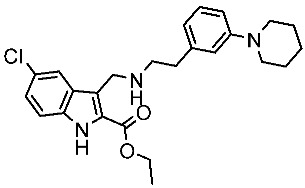	A549 MCF-7 Panc-1 HT-29	27 nM 30 nM 29 nM 30 nM	EGFR = 68 nM EGFR^T790M^ =8.6 nM BRAF^V600E^ = 35 nM	[175]
St.56	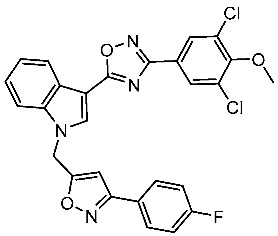	MCF-7 MDA-MB-231	3.12 µM 9.43 µM	EGFR = 311 nM	[176]
St.57	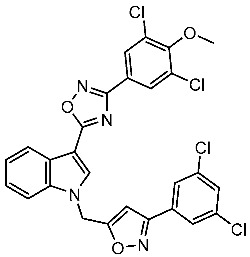	MCF-7 MDA-MB-231	2.16 µM 8.33 µM	EGFR = 203 nM	[176]
St.58	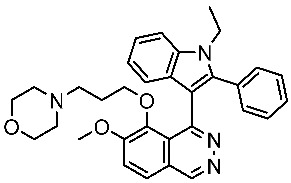	A549 PC-9 A431	4.1 µM 0.5 µM 2.1 µM	EGFR^L858R^ = 1.9 nM EGFR^wt^ = 5.2 nM	[178]

## 4. Challenges, Limitations, and Future Directions

While inhibitors targeting CDKs and EGFR have demonstrated substantial clinical success, significant challenges remain in maximizing their therapeutic potential. For CDKIs, the primary limitation has been the emergence of resistance mechanisms in cancer cells. Tumors can bypass CDK4/6 inhibition by activating alternative pathways, such as the PI3K/AKT/mTOR pathway, or through upregulation of cyclins and other regulatory proteins that restore cell cycle progression. Additionally, CDKIs often exhibit a narrow therapeutic window, resulting in dose-limiting toxicities, especially myelosuppression. Another limitation is that current CDKIs primarily concentricity on CDK4/6, while the roles of other CDKs (e.g., CDK7, CDK9) in cancer progression remain underexplored.

In the case of EGFR inhibitors, one of the biggest challenges is the development of acquired resistance due to secondary mutations in the EGFR gene, such as the T790M mutation in non-small cell lung cancer (NSCLC), which limits the long-term efficacy of first- and second-generation EGFR inhibitors. Furthermore, while EGFR inhibitors have been effective in certain EGFR-mutant cancers, they often show limited efficacy in cancers with wild-type EGFR or in tumors that develop compensatory signaling pathways, like MET amplification or activation of HER2 kinases. Off-target effects leading to toxicity, particularly in skin and gastrointestinal tissues, also remain a concern with EGFR inhibitors.

To enhance the selectivity of both CDKIs and EGFR inhibitors, more research is needed in the areas of structural biology and precision medicine. High-resolution structures of CDK and EGFR complexes, coupled with computational modeling, will aid in designing next-generation inhibitors with greater specificity and fewer side effects. A promising direction for both CDKIs and EGFR inhibitors involves exploring their role in combination with immunotherapies, as kinase inhibitors can modulate the tumor microenvironment, potentially improving immune responses to cancer. Ultimately, overcoming the resistance mechanisms and improving selectivity will be pivotal in realizing the full potential of these therapies.

## 5. Conclusions

In conclusion, the field of protein kinase inhibitors has significantly advanced cancer therapy by targeting key regulators of cellular processes. With 69 therapeutics approved by the FDA targeting approximately 24 protein kinases, these inhibitors have become vital in the treatment of neoplastic diseases. This review highlighted the progress in targeting CDKs and EGFR as promising strategies in cancer therapy. CDKs, particularly CDK4 and CDK6, have shown great promise with ATP-competitive inhibitors like palbociclib, abemaciclib, and ribociclib, leading to significant breakthroughs in metastatic breast cancer treatment. The introduction of ATP non-competitive inhibitors opens new avenues for expanding the therapeutic potential and discovering novel pharmacological properties. Future research will focus on refining combination therapies, identifying biomarkers for patient stratification, and understanding resistance mechanisms to enhance the effectiveness and tolerability of CDK inhibitors. Similarly, EGFR tyrosine kinase inhibitors (TKIs) have improved survival rates in patients with EGFR mutations, although resistance due to epigenetic mutations such as T790M poses a significant challenge. Innovative strategies, including the development of next-generation TKIs, multi-target agents, and combination therapies with other signaling pathway inhibitors, hold promise for overcoming resistance and enhancing treatment efficacy. Overall, the ongoing advancements in kinase inhibitor research underscore the importance of continuous exploration and innovation. By focusing on specific mutations, combination therapies, and personalized treatment approaches, future kinase inhibitors are poised to provide more effective and tailored cancer treatments, ultimately improving patient outcomes.

## Figures and Tables

**Table 1:** The FDA-approved CDK4/6 inhibitors’ structure, the year of approval, and therapeutic indications.

Drug Name	Chemical Structure	Year	Therapeutic Indications
Palbociclib	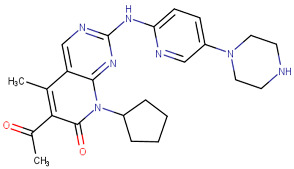	2015	Breast cancer combination therapy [56]
Abemaciclib	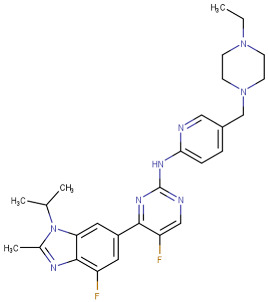	2017	Breast cancer [57]
Ribociclib	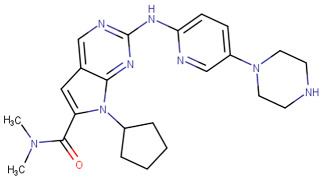	2017	Breast cancer combination therapy [58]
Trilaciclib	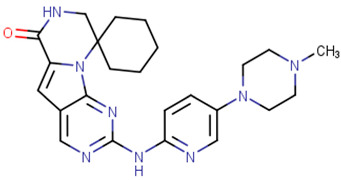	2021	Myeloprotective agent [59]

**Table 2:** The biological roles of CDKs and their correlation with various forms of cancer.

CDKs	Cyclin Partner	Biological Role	Type of Cancer	Ref.
CDK1	B	Regulate M phase	Breast, ovarian, lung	[65,66,67]
CDK2	A	Regulate G1-S phases, Rb-E2F pathway	Breast, ovarian, lung, prostate and many others	[62,68]
	E			
CDK4	D	Regulate G1 phase, Rb-E2F pathway	Skin, breast, bladder, lung	[69,70]
CDK6	D	Regulate G1 phase, Rb-E2F pathway	Bladder, esophageal, gastric, head and neck, pancreatic	[67,71]
CDK9	T	DNA damage repair and RNAPII transcription	Breast, lung, cervical, and many others	[72,73]

**Table 5:** The structures and the IC_50_ values against a panel of cancer cell lines and CDK4/6 for the most active agents of pyrimidine derivatives.

Code	Structure	Evaluated Cancer Cell Lines		CDK4	CDK6	Ref.
		Cell Lines	IC_50_	IC_50_		
St.26	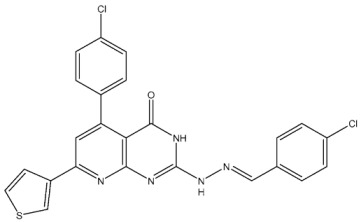	MCF-7 A549 PC-3	1.59 µM 2.48 µM 0.01 µM	-	115.38 nM	[113]
St.27	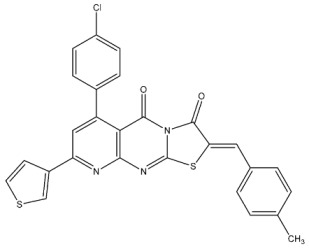	MCF-7 A549 PC-3	0.01 µM 1.69 µM 1.37 µM	-	726.2 nM	
St.28	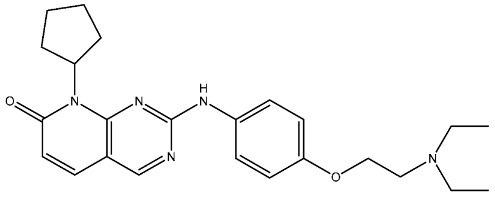	NA	NA	pIC_50_ = 9.15		[114]
St.29	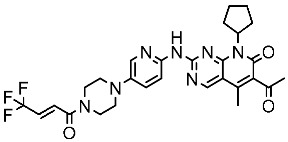	HepG2 A549 MDA-MB-231 MCF-7	5.10 µM 1.55 µM 0.51 µM 0.48 µM	18 nM	13 nM	[115]
St.30	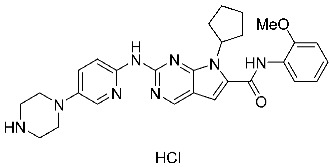	PaCa-2 BxPc-3	1.83 µM 4.12 µM	20.5 nM	52.3 nM	[116]
St.31	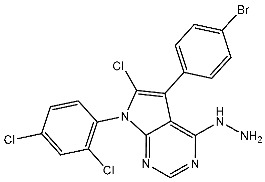	MCF-7 HepG2	1.70 µM 37.9 µM	Down-regulation	NA	[117]
St.32	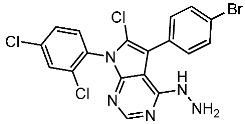	-	-	35.0 nM	318 nM	[118]
St.33	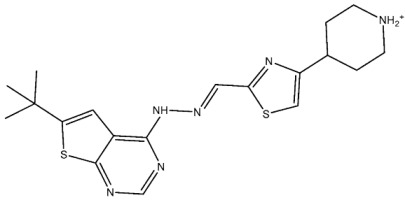	-	-	14 nM	-	[119]

**Table 6:** The structures and the IC_50_ values against a panel of cancer cell lines and CDK4/6 for the most active agents of miscellaneous-based derivatives.

Code	Structure	Evaluated Cancer Cell Lines		CDK4	CDK6	Ref.
		Cell Lines	IC_50_ or Inh.%	IC_50_		
St.34	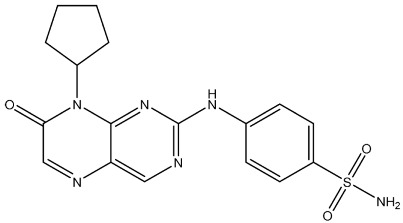	HCT116 MDA-MB-231 HeLa HT-29	0.65 µM 0.39 µM 0.70 µM 2.53 µM	34 nM	56.1 nM	[120]
St.35	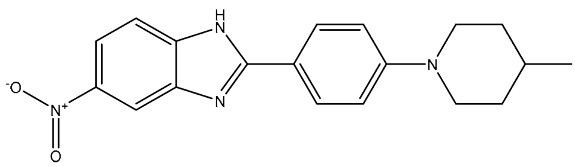	HL-60 NCIH522 HCT-15 PC-3 MCF-7	99.52% 101.83% 93.85% 79.80% 71.81%	-	172 nM	[121]
St.36	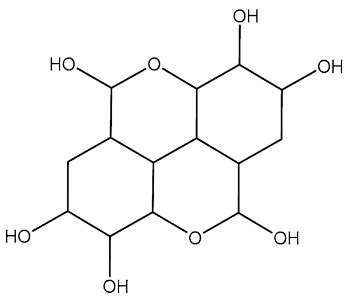	-	-	3.05 µM		[122]

**Table 7:** The structures and the IC_50_ values against a panel of cancer cell lines and CDK9 for the most active agents.

Code	Structure	Evaluated Cancer Cell Lines		CDK9	Ref.
		Cell Lines	IC_50_	IC_50_	
St.37	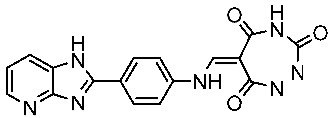	MCF-7 HCT116	0.63 µM 5.30 µM	0.50 µM	[132]
St.38	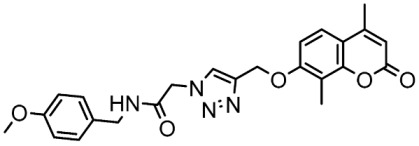	MCF-7 MDA-MB231 HCT116 WI38	3.47 µM 1.43 µM 12.56 µM 31.96 µM	0.38 µM	[133]
St.39	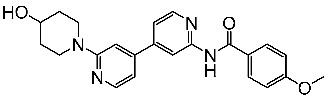	HCT116 HT-29 RKO SW480 DLD1	0.69 µM 0.88 µM 0.97 µM 0.99 µM 0.71 µM	30 nM	[134]
St.40	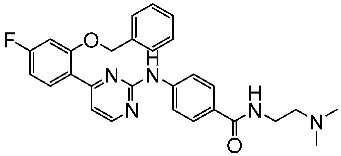	A549 H1975 A431 PANC-1 HCT116 LO2	0.66 µM 0.43 µM 0.10 µM 0.08 µM 0.09 µM 1.43 µM	10.4 nM	[135]
St.41	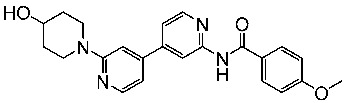	MDA-MB BT549 HepG2 HeLa A549	0.044 µM 0.147 µM 0.392 µM 0.158 µM 2.30 µM	>1 µM	[136]
St.42	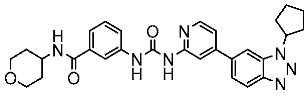	HCT116 HT-29	0.21 nM 0.49 nM	2.43 nM	[137]

**Table 8:** The FDA-approved EGFR inhibitors’ structure, the year of approval, mechanism of action, and therapeutic indications.

Drug Name	Chemical Structure	Year	MOA	Therapeutic Indications
Gefitinib	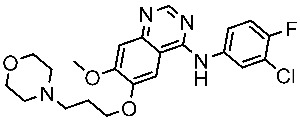	2003	Inhibits intracellular phosphorylation of numerous tyrosine kinases associated with transmembrane cell surface receptors	NSCLC [148,149]
Erlotinib	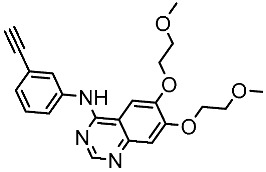	2004	Targets the EGFR tyrosine kinase	NSCLC and pancreatic cancer [150,151]
Lapatinib	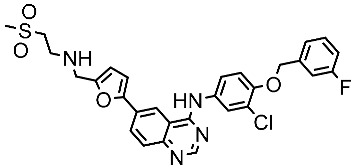	2007	Inhibits intracellular tyrosine kinase domains of both EGFR (ErbB1) and HER2	Breast cancer [152]
Osimertinib	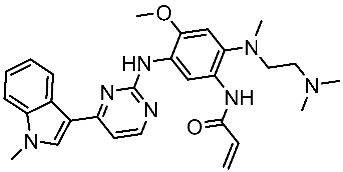	2015	Irreversible inhibits the epidermal growth (ErbB)	NSCLC [153]
Neratinib	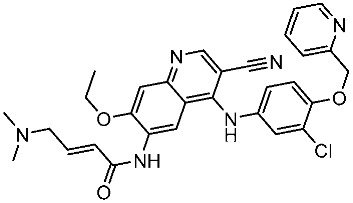	2017	Irreversible inhibitors of the receptor tyrosine kinase (RTK), ErbB2, RTK, and EGFR.	Breast cancer [154]
Dacomitinib	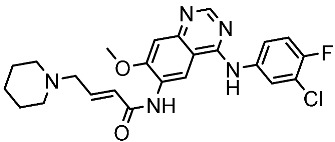	2018	Irreversible pan inhibitor of HER tyrosine kinases	Metastatic NSCLC [155]
Tucatinib	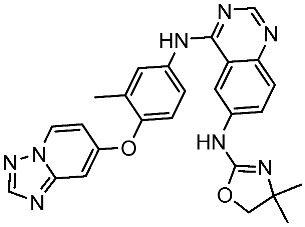	2020	Inhibits ERBB2 (HER2)	Breast cancer and colon cancer [156]
Mobocertinib	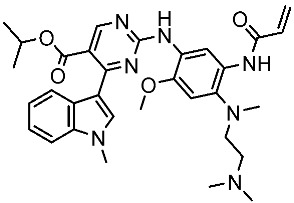	2021	Inhibits EGFR/HER2	NSCLC [157]

**Table 9:** The structures and the IC_50_ values against a panel of cancer cell lines and EGFR subtypes for the most active agents of quinazoline-based derivatives.

Code	Structure	Evaluated Cancer Cell Lines		EGFR	Ref.
		Cell Lines	IC_50_	IC_50_	
St.43	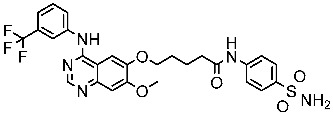	A549 A431 H1975	6.54 µM 4.04 µM 1.94 µM	EGFR^Wt^ = 27 nM EGFR^T790M^ = 9.2 nM	[160]
St.44	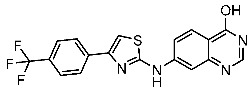	MCF-7 HepG2 A549	2.86 µM 5.91 µM 14.79 µM	2.17–3.62 nM	[161]
St.45	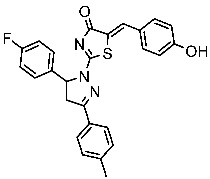	A549 T-47D	4.41 µM 1.15 µM	83 nM	[162]
St.46	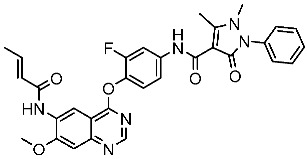	A549 H1975 PC-9	1.06 µM 1.13 µM 1.05 µM	EGFR^Wt^ = 99.8nM EGFR^L858R^ = 68.1 nM c-Met = 0.26 nM	[163]
St.47	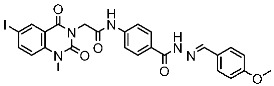	HepG2 MCF-7 HCT116 A549	5.70 µM 7.15 µM 5.76 µM 6.50 µM	EGFR^T790M^ = 0.30 µM VEGFR-2 = 0.90 µM	[164]
St.48	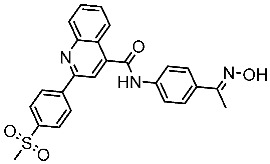	Panc-1 MCF-7 HT29 A549	1.50 µM 1.10 µM 1.60 µM 1.30 µM	EGFR = 105 nM BRAF^V600E^ = 140 nM	[165]

**Table 10:** The structures and the IC_50_ values against a panel of cancer cell lines and EGFR subtypes for the most active agents of pyrimidine-based derivatives.

Code	Structure	Evaluated Cancer Cell Lines		EGFR or Related	Ref.
		Cell Lines	IC_50_	IC_50_	
St.49	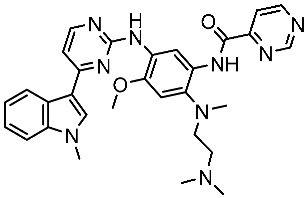	A549 H1975	2.53 µM 1.56 µM	EGFR^Wt^ =2 nM EGFR ^L858R/T790M^ = 10 nM	[168]
St.50	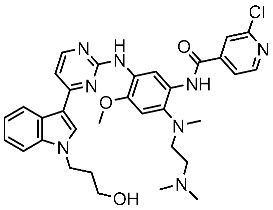	A549 H1975	2.14 µM 1.82 µM	EGFR^Wt^ = 743 nM EGFR ^L858R/T790M^ = 42 nM	[168]
St.51	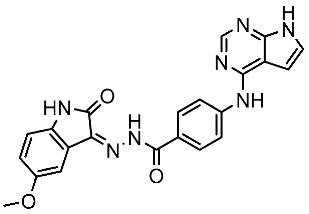	HeLa MCF-7 HepG2 MDA-MB-231	1.98 µM 5.93 µM 6.11 µM 2.48 µM	EGFR = 103 nM VEGFR-2 = 178 nM CDK2 = 131 nM HER2 = 81 nM	[169]
St.52	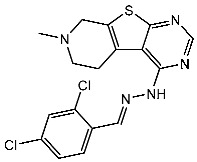	HL-60 K-562 HOP92 COLO205 SNB-75 LOX MDA-MB-468	2.12 µM 2.08 µM 1.95 µM 1.66 µM 1.54 µM 1.49 µM 1.52 µM	77 nM	[170]
St.53	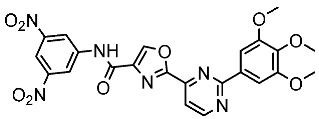	PC3 A549 HepG2 MDA-MB-468 DU-145	0.13 µM 0.15 µM 0.11 µM 0.13 µM 0.10 µM.	EGFR^Wt^ = 60 nM EGFR^T790M^ = 72 nM	[171]

## Data Availability

This is a Review article and there are no new data in this manuscript.

## Abbreviations

CDK	cyclin-dependent kinase
CDKI	cyclin-dependent kinase inhibitor
EGFR	epidermal growth factor receptor
VEGFR	vascular endothelial growth factor receptor
FDA	the Food and Drug Administration of the United States
FGFR	fibroblast growth factor receptor
HER2	human epidermal growth factor receptor-2
NSCLC	non-small cell lung cancer
PDGFR	platelet-derived growth factor receptor
ATP	adenosine triphosphate
HDAC	Histone deacetylase
IC_50_	inhibitory concentration
GI_50_	half-maximum growth inhibition
PDB	Protein Data Bank
SAR	structure-activity relationship
WHO	World Health Organization
M phase	mitosis phase
RTK	receptor tyrosine kinase
MOA	Mechanism of action
**Cancer Cell Lines Explanation**	
A375	melanoma
A431	squamous
MDA-MB231	breast
Colo205	colon
HCT116	colon
A549	lung
MCF-7	breast
PC3	prostate
HepG2	liver
MDA-MB-468	breast
T-47D	breast
HeLa	cervical
K-562	leukemia
A2780	ovarian
HL60	leukemia
HT-29	colon
MOLT-4	lymphoblastic leukemia
H460	lung
SMMC77	liver
MV4-11	leukemia
SKOV3	ovarian
WI-38	lung
Panc-1	pancreas
PaCa-2	pancreas
BxPc-3	pancreas
NCIH522	lung
HCT-15	colon
RKO	colon
SW480	colon
DLD1	colon
LO2	liver
PC-9	lung
HOP92	lung
SNB-75	brain
DU-145	brain
